# PAT4 levels control amino-acid sensitivity of rapamycin-resistant mTORC1 from the Golgi and affect clinical outcome in colorectal cancer

**DOI:** 10.1038/onc.2015.363

**Published:** 2015-10-05

**Authors:** S-J Fan, C Snell, H Turley, J-L Li, R McCormick, S M W Perera, S Heublein, S Kazi, A Azad, C Wilson, A L Harris, D C I Goberdhan

**Affiliations:** 1 Department of Physiology, Anatomy and Genetics, University of Oxford, Oxford, UK; 2 Molecular Oncology Unit, Weatherall Institute of Molecular Medicine, University of Oxford, Oxford, UK

## Abstract

Tumour cells can use strategies that make them resistant to nutrient deprivation to outcompete their neighbours. A key integrator of the cell's responses to starvation and other stresses is amino-acid-dependent mechanistic target of rapamycin complex 1 (mTORC1). Activation of mTORC1 on late endosomes and lysosomes is facilitated by amino-acid transporters within the solute-linked carrier 36 (SLC36) and SLC38 families. Here, we analyse the functions of SLC36 family member, SLC36A4, otherwise known as *p*roton-assisted *a*mino-acid *t*ransporter 4 (PAT4), in colorectal cancer. We show that independent of other major pathological factors, high PAT4 expression is associated with reduced relapse-free survival after colorectal cancer surgery. Consistent with this, PAT4 promotes HCT116 human colorectal cancer cell proliferation in culture and tumour growth in xenograft models. Inducible knockdown in HCT116 cells reveals that PAT4 regulates a form of mTORC1 with two distinct properties: first, it preferentially targets eukaryotic translation initiation factor 4E-binding protein 1 (4E-BP1), and second, it is resistant to rapamycin treatment. Furthermore, in HCT116 cells two non-essential amino acids, glutamine and serine, which are often rapidly metabolised by tumour cells, regulate rapamycin-resistant mTORC1 in a PAT4-dependent manner. Overexpressed PAT4 is also able to promote rapamycin resistance in human embryonic kidney-293 cells. PAT4 is predominantly associated with the Golgi apparatus in a range of cell types, and *in situ* proximity ligation analysis shows that PAT4 interacts with both mTORC1 and its regulator Rab1A on the Golgi. These findings, together with other studies, suggest that differentially localised intracellular amino-acid transporters contribute to the activation of alternate forms of mTORC1. Furthermore, our data predict that colorectal cancer cells with high PAT4 expression will be more resistant to depletion of serine and glutamine, allowing them to survive and outgrow neighbouring normal and tumorigenic cells, and potentially providing a new route for pharmacological intervention.

## Introduction

During cancer growth, tumour cell adaptation is driven by adverse microenvironmental conditions such as hypoxia and starvation.^1^ Mechanistic target of rapamycin complex 1 (mTORC1) responds to both local nutrient status and growth factor signalling through phosphatidylinositol 3-kinase to regulate protein synthesis and cellular homeostasis, thereby modulating cancer cell growth, metabolism and metastasis.^2, 3, 4^ However, attempts to block tumour growth using the allosteric mTOR inhibitor rapamycin or its analogues have met with limited success.^5^ Although these drugs strongly reduce signalling to one of the two well-characterised mTORC1 targets, ribosomal protein p70-S6 kinase 1 (S6K1), they often have more limited effects on the other, eukaryotic translation initiation factor 4E-binding protein 1 (4E-BP1), a negative regulator of eukaryotic initiation factor 4E (eIF4E)^6, 7^ implicated in metastatic growth.^8, 9^ This resistance can sometimes be circumvented by using adenosine triphosphate (ATP)-competitive mTOR inhibitors,^5, 6, 7^ which also block the other mTOR kinase-containing complex, mTORC2. Nonetheless, how changes in mTOR structure^10^ or mTOR regulators modulate rapamycin sensitivity remains of considerable interest.

Members of the *p*roton-assisted *a*mino-acid *t*ransporter (PAT) or solute-linked carrier 36 (SLC36) family^11^ were identified as positive regulators of growth and mTORC1 signalling through an *in vivo* genetic overexpression screen in flies.^12, 13^ These effects were shown to be conserved by characterisation of the two ubiquitously transcribed human *PAT*s, *PAT1* (*SLC36A1*) and *PAT4* (*SLC36A4*).^14^ The prototypic PAT family member, PAT1, is a lysosomal amino-acid transporter (AAT).^15, 16^ In rapidly growing cells, it is located at the surface of nutrient-rich late endosomal and lysosomal (LEL) compartments,^13^ where mTOR accumulates in response to amino-acid stimulation. The recruitment of mTOR requires assembly of a multiprotein complex, which includes Raptor, a heterodimeric pair of Ras-related Rag GTPases, the pentameric Ragulator, and the vacuolar-H^+^-ATPase proton pump at the compartment surface (reviewed in Bar-Peled and Sabatini^2^ and Malik *et al.*^2, 3^). PAT1 also interacts with this complex to promote mTOR localisation on LELs and subsequent mTORC1 signalling. Amino-acid sensing by the PATs may involve transport or signalling via a so-called ‘transceptor' mechanism.^4, 13, 17^

Recent studies have identified an AAT in the related SLC38 family, SLC38A9, which also interacts on LELs with the mTORC1-regulatory machinery, potentially in response to arginine,^18, 19^ suggesting that different LEL-located, mTORC1-regulatory AATs may sense different amino acids. Furthermore, the identification of molecules such as Golgi-localised Rab1A,^20^ and ADP ribosylation factor Arf1^21^ and phospholipase D^22, 23^ as regulators of Rag-independent, mTORC1 activation, suggests that other amino-acid-sensing mechanisms remain to be discovered.

Here we investigate PAT4 function in colorectal cancer. Colorectal cancers are frequently rapamycin-resistant^6^ and often metastatic, seriously impacting on clinical outcome.^24, 25^ We show that PAT4 upregulation is associated with cancer progression. By using an inducible *PAT4* shRNA knockdown in HCT116 colorectal cancer cells, we find that PAT4 responds to two rapidly metabolised, non-essential amino acids, glutamine and serine,^26, 27^ to drive rapamycin-resistant, mTORC1-mediated cell proliferation. Furthermore, we provide evidence that PAT4 interacts with Rab1A and mTORC1 on the Golgi, suggesting a role in amino-acid-sensing from this compartment.

## Results

### Validation of a novel PAT4 monoclonal antibody

**Figure 1:**
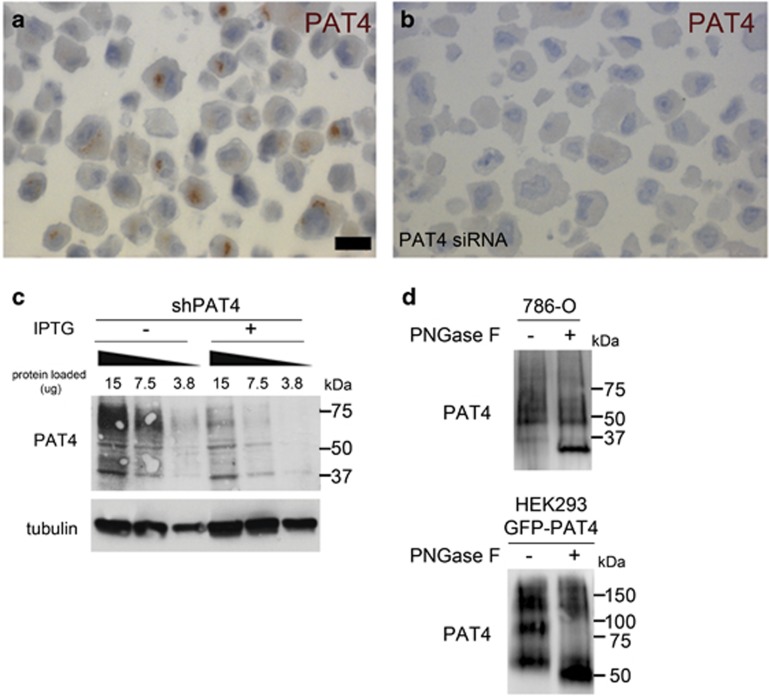
Validation of an ‘in-house'-generated anti-PAT4 monoclonal antibody. (**a** and **b**) Formalin-fixed, paraffin-embedded 786-O cells incubated with PAT4 monoclonal antibody (Pat4/9/H10) and visualised with 3,3'-diaminobenzidine (DAB). There is an obvious perinuclear region of staining visible in most cells treated with the scrambled control siRNA (**a**). This staining is absent when the *PAT4* transcript is knocked down using an siRNA against *PAT4* (si435; Heublein *et al.*;^14^ **b**). (**c**) Western blot analysis of serial dilutions of cell lysates (15, 7.5 and 3.8 μg of protein) produced from pools of 786-O cells carrying an IPTG-inducible shPAT4 (43587; shPAT4) probed with PAT4 monoclonal antibody Pat/9/H10. This reveals a set of bands from 60–75 kDa that is strongly reduced by IPTG-induced *PAT4* knockdown (+IPTG), suggesting that they are PAT4-specific. Western blots were also probed with an anti-tubulin antibody as a loading control. (**d**) Western blot of cell lysates from 786-O cells and from a GFP-PAT4-overexpressing HEK-293 cell line treated with PNGase F before electrophoresis to remove glycosyl groups. This resolves the crossreacting molecules seen in untreated cell lysates into more specific bands migrating at ~30 and 50 kDa, respectively, smaller than the predicted molecular weights of 55 kDa (PAT4) and 85 kDa (GFP-PAT4), a phenomenon also reported for other transmembrane proteins.^43^

We generated a highly specific mouse monoclonal antibody against PAT4 (antibody Pat4/9/H10). Staining with this antibody revealed that PAT4 was localised to an asymmetric perinuclear region in formalin-fixed, paraffin-embedded 786-O renal cancer cells, which express high PAT4 levels, and lost in 786-O cells transfected with *PAT4* small interfering RNA (siRNA) (Figures 1a and b). Bands of 60–75 kDa molecular weight were observed on western blots of cell lysates (Figure 1c) and strongly reduced after *PAT4* knockdown, confirming antibody specificity. This smear resolved into a band of ~30 kDa after pretreatment of lysates with the glycosidase, peptide-N-glycosidase F (PNGase F), smaller than the predicted 55 kDa molecular weight (Figure 1d).

### High PAT4 expression is associated with poor outcome in colorectal cancer patients

**Figure 2:**
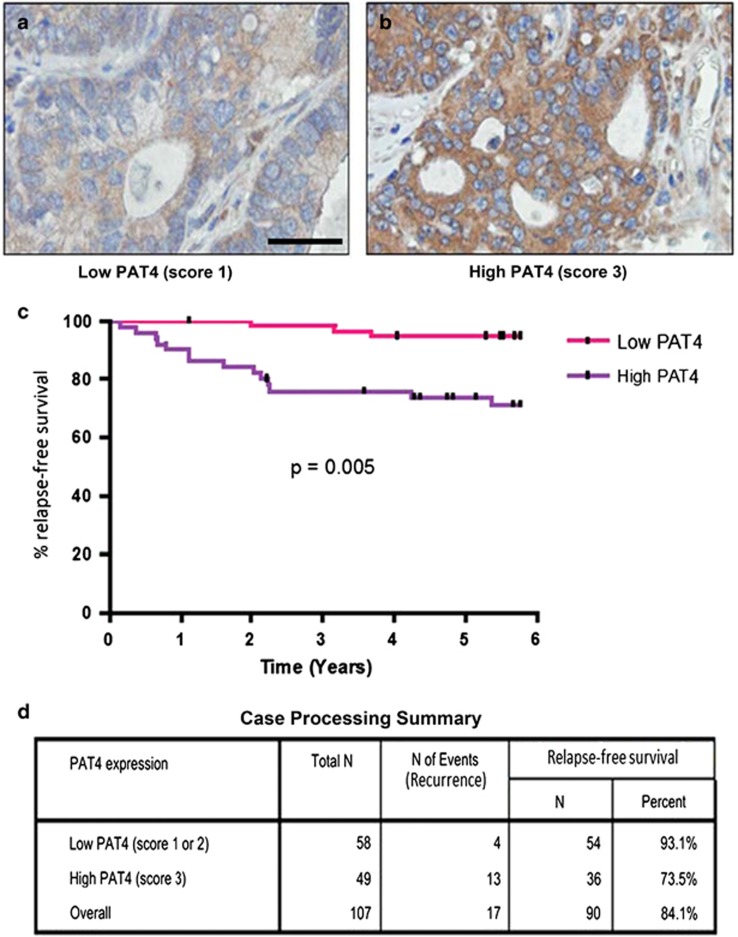
High expression of PAT4 predicts poorer relapse-free survival in colon cancer. In all, 107 patients with primary colonic carcinoma were assessed for expression of PAT4 in their tumours and stratified according to PAT4 expression. (**a** and **b**) Representative images of low and high PAT4 expression levels as determined by immunohistochemistry. Brown staining with diaminobenzidine indicates immunoreactivity. (**c**) Kaplan–Meier curves compare high versus low levels of expression. *P*-value is the result of a log-rank test (Mantel–Cox). (**d**) Case processing summary for high and low PAT4-expressing patients. The scale bar in (**a**) is 50 μm.

To test whether PAT4 expression is altered in human colorectal cancer, we stained primary tumour tissue microarrays from 107 patients, who had been treated by surgical resection only. The intensity of cytoplasmic staining was scored by a pathologist (CS) into three categories (Figures 2a and b; see Materials and methods), all of which were higher than normal colorectal epithelium. Statistical analysis showed no association between high PAT4 expression and standard clinical or pathological variables, including site of tumour, tumour stage, nodal or distal metastases, age, lymphatic, vascular or neural invasion, differentiation or gender (Supplementary Table S1).

In univariate analyses, high PAT4 levels (*P*=0.01) as well as high tumour stage (*P*<0.01), tumour stage score (*P*<0.01), the presence of bowel perforation (*P*=0.02), neural invasion (*P*<0.01), nodal (*P*<0.01) and synchronous metastasis (*P*<0.01) significantly correlated with shorter relapse-free survival (Supplementary Table S2). Patients with cancers that had higher PAT4 expression had a significantly shorter mean relapse-free survival compared with those with lower levels (*P*<0.01; Figures 2c and d). Additionally, higher PAT4 levels showed statistical significance in multivariate survival analysis (*P*<0.01; Supplementary Table S3). The multivariate model included all variables significantly associated with relapse in univariate analysis, apart from overall stage, as this is calculated from tumour stage (T), nodal metastases (N) and distant metastases (M) stages. We conclude that increased PAT4 levels are associated with worse prognosis in patients with colorectal cancer.

### PAT4 regulates HCT116 cell proliferation

**Figure 3:**
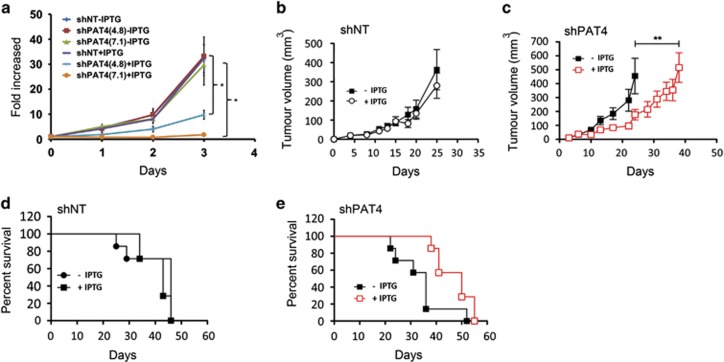
PAT4 regulates the growth of HCT116 cells *in vitro* and *in vivo*. (**a**) Proliferation of clones of HCT116 cells stably transduced with one of two independent IPTG-inducible shRNA constructs targeting PAT4, namely shPAT4(4.8) and shPAT4(7.1), or the IPTG-inducible non-targeting control construct (shNT) was measured in the presence and absence of IPTG (*n*=3). (**b** and **c**) Mean growth curves (±s.e.m.) of human HCT116 tumour xenografts in immunodeficient mice carrying pools of cells transduced with shNT (**b**) with (empty circles) or without (filled squares) IPTG induction, or shPAT4-transduced HCT116 cells (**c**) with (empty squares, outlined in red) or without (filled squares) IPTG induction (*n*=7). Data in (**b** and **c**) were analysed by unpaired two-tailed independent Student's *t*-test. (**d** and **e**) Kaplan–Meier survival curves of seven animals with and without IPTG induction of shNT- (**d**) and shPAT4-containing (**e**) HCT116 tumours; log-rank (Mantel–Cox) test: *P*=0.019 for (**e**). For shPAT4-inducible cells, all but one of the seven non-induced mice (filled squares) needed to be killed within 36 days (median survival time of 36 days), whereas all seven induced mice (empty squares, outlined in red) were killed from 38 days onwards with a median survival time of 50 days. Cell proliferation experiment was repeated three times. *P<0.05, **P<0.01.

To analyse PAT4 function in HCT116 colorectal cancer cells, we generated stably transduced cell lines, each carrying one of three different lentiviral constructs expressing a *PAT4* short hairpin RNA (shRNA) under isopropyl β-d-1-thiogalactopyranoside (IPTG)-inducible control. For each construct, pooled cells representing many individual transduction events had reduced *PAT4* transcript levels, as determined by quantitative real-time PCR (Supplementary Figure S1A). Two shRNAs, 49 384 and 49 387, were selected for further study. To induce a uniformly strong *PAT4* knockdown, single-cell clones were isolated from another shRNA transduction, and named shPAT4(4.8) and shPAT4(7.1), respectively (Supplementary Figure S1B). *In vitro* culture of these clones together with HCT116 cells containing an IPTG-inducible, non-targeting shRNA (*shNT*) gene revealed that IPTG induction specifically inhibited proliferation of shPAT4-expressing cells (*P*<0.05; Figure 3a) with no significant effect on cell death (Supplementary Table S4).

### PAT4 promotes human tumour growth in xenograft models

To assess the role of PAT4 in tumour growth, pooled clones of HCT116 cells carrying shPAT4(49 387), were used in xenograft experiments. We reasoned that the variable level of *PAT4* knockdown in these cells might better model changes taking place in heterogeneous tumours expressing different levels of PAT4. The effect on tumour growth of IPTG-induced *PAT4* knockdown in shPAT4 HCT116 cells was assessed in mice over a 60-day period. Immunodeficient mice were provided with IPTG in their drinking water to induce shRNA expression. IPTG did not alter the size of tumours formed from shNT cells (Figure 3b). However, IPTG-induced shPAT4 expression reduced tumour growth significantly compared with non-induced shPAT4 controls (Figure 3c; *P*<0.01). In addition, shPAT4 induction extended median survival time of mice from 36 to 50 days (ratio: 0.72; 95% confidence interval of ratio: 0.36–1.07; Gehan–Breslow–Wilcoxon Test, *P*=0.008; Figures 3d and e). Induction of *PAT4* knockdown in a second experiment also significantly reduced mean tumour volume (*P*<0.05) and improved animal survival (*P*<0.05), demonstrating that PAT4 promotes HCT116 tumour growth *in vivo*.

### PAT4 regulates a rapamycin-resistant form of mTORC1

**Figure 4:**
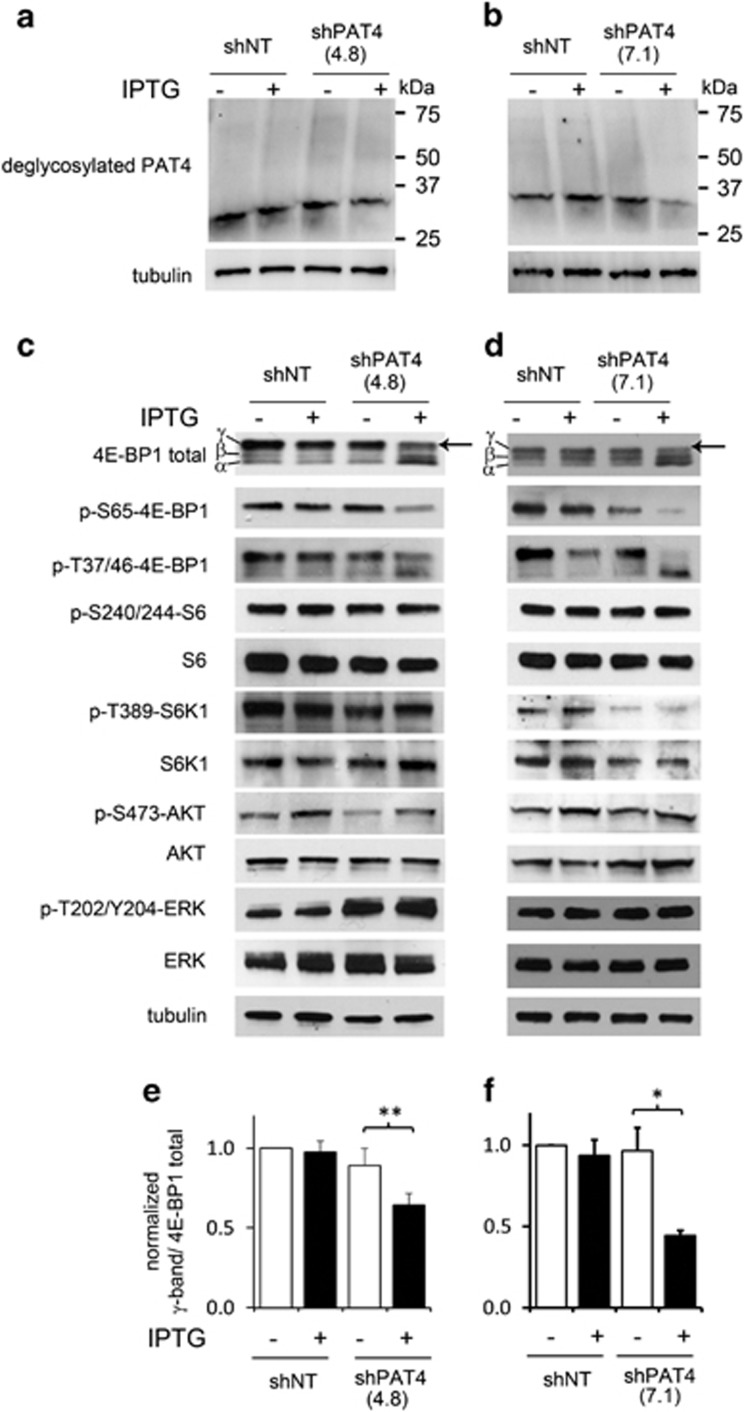
PAT4 selectively affects the mTORC1 target 4E-BP1. Western blots of protein extracts from HCT116 cells carrying either IPTG-inducible shNT or shPAT4(4.8) (**a** and **c**) or shNT and shPAT4(7.1) constructs (**b** and **d**) cultured in the presence and absence of IPTG. (**a** and **b**) *PAT4* knockdown reduces the level of PAT4 protein detected following incubation of cell lysates with PNGase F before electrophoresis. (**c**–**f**) *PAT4* knockdown significantly reduced the level of the most phosphorylated γ-form of 4E-BP1, visualises both with an anti-phospho-Ser65-4E-BP1 (p-S65-4E-BP1) antibody and also as the upper band with a pan-4E-BP1 antibody (arrow; quantified in three independent experiments as a proportion of total 4E-BP1 staining in histograms in (**e**) and (**f**), respectively). Phospho-S6K (p-T389-S6K1), phospho-S6 (p-S240/244-S6), phospho-Akt (p-S473-AKT) and phospho-ERK (p-T202/Y204-ERK) levels are essentially unaffected by *PAT4* knockdown. Blots were probed with an anti-tubulin antibody as a loading control. Effects were reproduced in three separate experiments. *P<0.05; **P<0.01.

To determine how *PAT4* knockdown might inhibit tumour growth, we analysed mTORC1 signalling in stably transfected HCT116 clones carrying inducible *PAT4* shRNAs, shPAT4(4.8) and shPAT4(7.1), and in the non-targeting clone, shNT. PAT4 protein is expressed at much lower levels in HCT116 cells compared with 786-O cells, thus lysates from large pools of HCT116 cells were pretreated with PNGase F to detect PAT4, which resolved into a specific 30 kDa band that was clearly reduced after IPTG addition in knockdown cells (Figures 4a and b).

**Figure 5:**
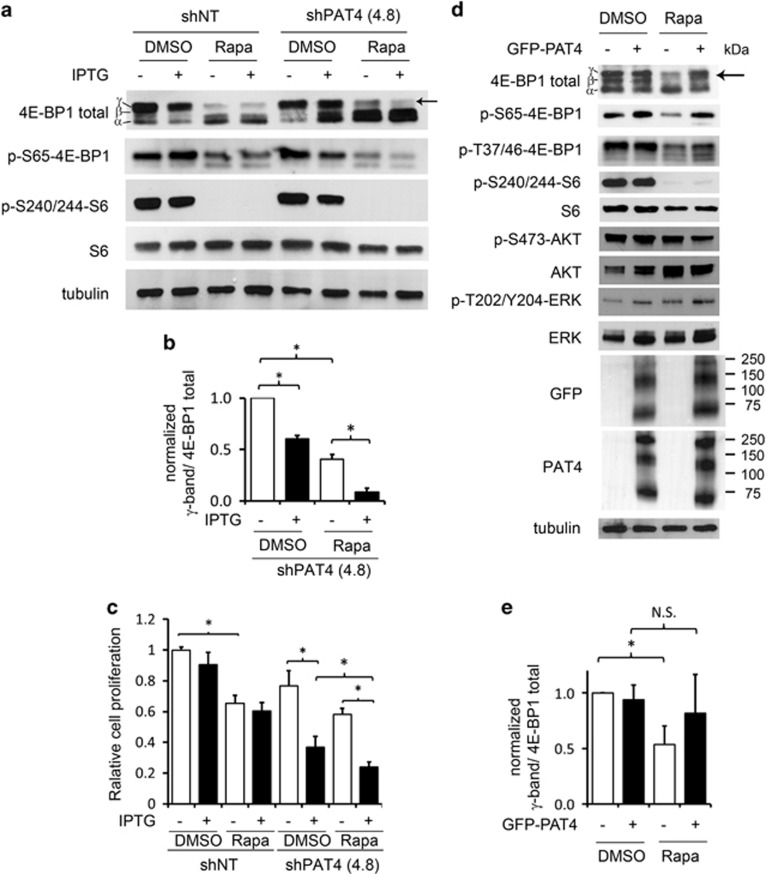
PAT4 regulates a rapamycin-resistant form of mTORC1 in HCT116 cells and increases rapamycin resistance in HEK-293 cells. (**a** and **b**) Clones of HCT116 cells carrying the IPTG-inducible *PAT4* shRNA construct, shPAT4(4.8), and the IPTG-inducible non-targeting control construct, shNT, were cultured for 5 days in the absence or presence of IPTG and, if required, treated with rapamycin for the last 24 h. Rapamycin (3 nM; see Supplementary Figures S2A and B) strongly reduces phospho-S6 levels (p-S240/244-S6), and partially affects the Ser65-phosphorylated 4E-BP1 (p-S65-4E-BP1) γ-band (arrow). This rapamycin-resistant phospho-4E-BP1 γ-band is almost completely lost after *PAT4* knockdown (**b**). (**c**) shPAT4(4.8) cells and shNT controls were cultured for 8 days in the absence or presence of IPTG and treated with rapamycin for 3 days. Cells were then counted revealing reduced proliferation for both cell lines in the presence of rapamycin, and also for shPAT4(4.8) in the presence of IPTG (*n*=3). The combination of both rapamycin and IPTG leads to further reduction in the cell number of shPAT4(4.8). (**d** and **e**) Normal HEK-293 cells or cells stably transfected with a constitutively expressed GFP-PAT4 construct were cultured for 24 h in the presence or absence of 100 nM rapamycin (see Supplementary Figure S2F), and then cell lysates analysed by western analysis. GFP-PAT4 expression reduces the effect of rapamycin on the γ-phosphorylated 4E-BP1 band (**e**), but not phospho-S6 (p-S240/244-S6), phospho-Akt (p-S473-AKT) or phospho-ERK (p-T202/Y204-ERK). All blots were probed with an anti-tubulin antibody as a loading control. (*P<0.05; *n*=3). The cell proliferation experiment was repeated three times. NS, not significant.

IPTG-induced *PAT4* knockdown selectively reduced the most highly phosphorylated form of 4E-BP1, designated the γ-band on western blots,^28^ whereas levels of less phosphorylated forms increased (Figures 4c and f). Human 4E-BP1 has at least eight phosphorylation sites.^28^ Phosphorylation of 4E-BP1 at Ser65, a key residue for eukaryotic initiation factor 4E binding,^29^ is typically required to form the γ-band. An anti-phospho-Ser65-4E-BP1 antibody verified that phosphorylation of this residue (p-S65-4E-BP1) was strongly decreased by *PAT4* knockdown. In contrast, overall phosphorylation of p-T37/46-4E-BP1 was maintained, but distributed between multiple 4E-BP1 bands after knockdown. *PAT4* knockdown either had no effect on S6K1 (p-T389-S6K1) and S6 (p-S240/244-S6) phosphorylation (Figures 4c and d), or sometimes led to a modest reduction in p-S240/244-S6 (e.g., Figure 5a and Supplementary Figure S2C).

To confirm that *PAT4* knockdown was not altering 4E-BP1 phosphorylation by inhibiting upstream phosphatidylinositol 3-kinase/Akt signalling, levels of phosphorylated mTORC2-regulated Akt (p-S473-AKT) were assessed; no change was observed (Figures 4c and d). Phosphorylation of extracellular signal-regulated kinase (ERK) mitogen-activated protein kinase (p-T202/Y204-ERK) by oncogenic forms of KRAS has also been associated with resistance to mTOR kinase inhibitors in colorectal cancer.^30^ HCT116 cells carry an oncogenic KRAS-G13D allele, but *PAT4* knockdown did not reduce ERK phosphorylation (Figures 4c and d), suggesting that it does not act through ERK to regulate mTORC1.

The selective action of *PAT4* knockdown on 4E-BP1 phosphorylation versus the S6K/S6 signalling arm of the mTORC1 pathway is complementary to the effects reported with rapamycin in these cells,^6^ where 4E-BP1 γ-phosphorylation is particularly resistant to this drug. Even at rapamycin concentrations that essentially blocked S6 phosphorylation, residual 4E-BP1 γ-phosphorylation was observed, as well as lower-molecular-weight 4E-BP1 phosphorylated on Ser65 (Figure 5a and Supplementary Figures S2A and B). However, when rapamycin and *PAT4* knockdown were combined, the γ-form of phosphorylated 4E-BP1 and remaining p-S65-4E-BP1 bands were almost completely lost (Figures 5a and b and Supplementary Figures S2C and D). Unlike rapamycin, an ATP-kinase inhibitor of mTOR, PP242, strongly affected both 4E-BP1 γ-phosphorylation and S6K activity in HCT116 cells (Supplementary Figure S2E). Taken together, these findings suggest that in HCT116 cells essentially all 4E-BP1 γ-phosphorylation is mTORC1-dependent (see also Ducker *et al.*^30^), but that some phosphorylation is rapamycin-resistant and regulated by PAT4. Importantly, rapamycin and *PAT4* knockdown also had additive effects on HCT116 cell proliferation (Figure 5c), demonstrating they inhibit different mTORC1 complexes, which both have a role in cell proliferation.

To test the link between PAT4 expression and rapamycin resistance, we overexpressed a green fluorescent protein (GFP)-tagged form of PAT4 in HEK-293 cells. We have previously shown in these cells that siRNA-induced knockdown of PAT4 reduces both 4E-BP1 and S6K/S6 phosphorylation, and this was confirmed using shRNA knockdown (Supplementary Figure S3). We observed that 4E-BP1 hyperphosphorylation shows some rapamycin resistance in HEK-293 cells (Supplementary Figure S2F). Strong overexpression of GFP-PAT4, which produced several protein bands resolving to a 50 kDa band on western blots after PNGase pretreatment (Figure 1d), increased rapamycin resistance of hyperphosphorylated, p-Ser65-4E-BP1 (Figures 5d and e). This suggests that high PAT4 levels can induce resistance to rapamycin in multiple different cell types.

### PAT4-dependent sensitivity of rapamycin-resistant mTORC1 to glutamine and serine

**Figure 6:**
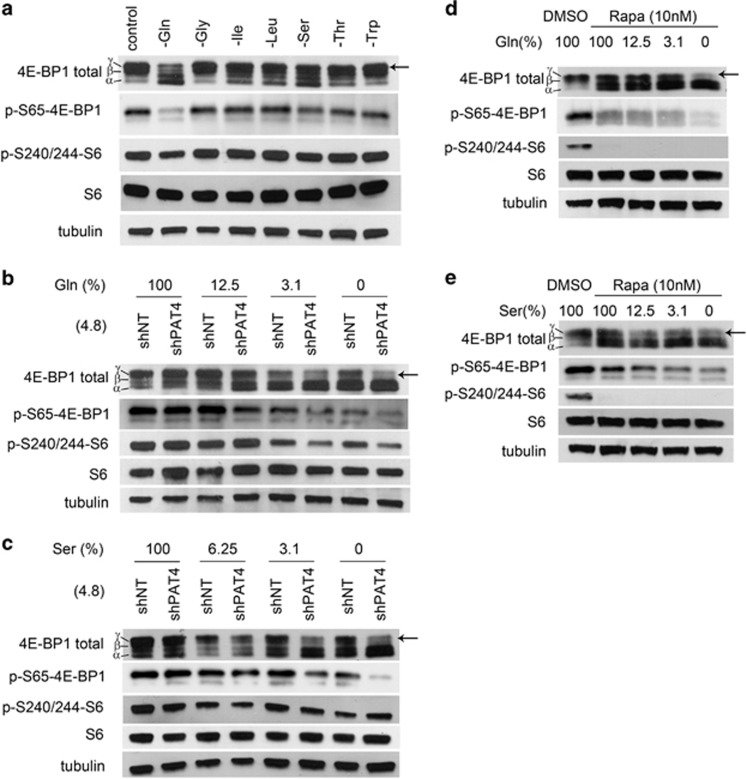
Glutamine and serine selectively regulate PAT4-dependent rapamycin-resistant mTORC1 signalling in HCT116 cells. (**a**) HCT116 cells were starved of the essential amino acids, glycine (Gly), isoleucine (Ile), leucine (Leu), threonine (Thr) and tryptophan (Trp), and the non-essential amino acids, serine (Ser) and glutamine (Gln), for 4 h in separate cultures, and then proteins extracted and subjected to western blotting. After this time, glutamine depletion produces the greatest reduction in γ-phosphorylation of 4E-BP1 (arrow); serine depletion also induces a more modest shift. (**b**) Clones of HCT116 cells carrying the IPTG-inducible *PAT4* shRNA constructs, shPAT4(4.8), and the IPTG-inducible non-targeting control construct, shNT, were exposed to culture medium containing different concentrations of glutamine for 4 h in the absence of IPTG. Under these conditions, shPAT4(4.8) cells express about 50% of normal *PAT4* mRNA levels (Supplementary Figure S1B) because of leaky *PAT4* shRNA transcription. As glutamine concentration falls, a greater reduction in γ-phosphorylation of 4E-BP1 is apparent in shPAT4(4.8) cells. S6 phosphorylation also seems to be affected at low glutamine concentrations. (**c**) Same experiment as (**b**), except different levels of serine in the culture medium. Again, shPAT4(4.8) cells have lower 4E-BP1 γ-phosphorylation at reduced concentrations of serine. (**d** and **e**) Rapamycin-treated HCT116 cells subjected to different levels of glutamine (**d**) and serine starvation (**e**), over 4 h, show a greater reduction in γ-phosphorylation of 4E-BP1.

As PATs are implicated in amino-acid-dependent mTORC1 activation, we hypothesised that PAT4 might sense levels of specific amino acids that regulate rapamycin-resistant mTORC1. We starved HCT116 cells of specific amino acids, including two non-essential amino acids required for HCT116 growth. Non-essential serine is diverted into glycolysis, whereas glutamine fuels the tricarboxylic acid cycle via glutaminolysis in cancer cells, including HCT116 cells.^26, 27, 31^ Reducing either amino acid, particularly glutamine, over 4 h had a stronger inhibitory effect on 4E-BP1 hyperphosphorylation compared with loss of any essential amino acid (Figure 6a).

If some of the selective effects of glutamine and serine on 4E-BP1 are mediated via a PAT4-dependent sensing mechanism, we reasoned that modest changes in PAT4 levels might alter the sensitivity of HCT116 cells to glutamine and serine starvation. Our *PAT4* knockdown clones displayed leaky IPTG-independent shRNA knockdown (Supplementary Figure S1B), which normally did not affect 4E-BP1 phosphorylation (Figure 5a) or proliferation (Figure 3a). We tested the amino-acid sensitivity of these cells in the absence of IPTG. 4E-BP1 hyperphosphorylation was more sensitive to reduction in glutamine and serine levels in both shPAT4(4.8) (Figures 6b and c) and shPAT4(7.1) (Supplementary Figures S4) clones, indicating that PAT4 is involved in the sensing of these metabolically important amino acids. Interestingly, S6 phosphorylation also appeared to be more sensitive to reduced glutamine and serine in these experiments, suggesting either that rapamycin-resistant mTORC1 can directly or indirectly modulate S6K activity under certain conditions or that starvation affects the specificity of PAT4 for rapamycin-resistant mTORC1. Combining glutamine or serine starvation with rapamycin treatment had a greater effect on 4E-BP1 hyperphosphorylation compared with either treatment alone (Figures 6d and e), supporting our conclusion that these amino acids are sensed by PAT4-regulated, rapamycin-resistant mTORC1.

### Golgi-localised PAT4 interacts with mTORC1

**Figure 7:**
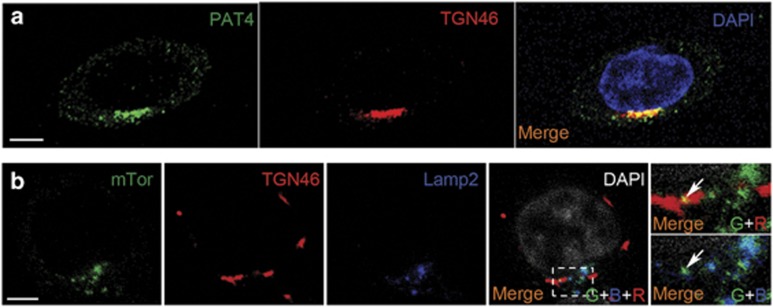
PAT4 and mTOR are located on the Golgi in HCT116 cells. (**a**) Endogenous PAT4 (green) is expressed in compartments that include the *trans*-Golgi network (TGN46; red) in HCT116 cells. (**b**) Although most mTOR (green) is located on LAMP2-positive late endosomes and lysosomes (blue) in HCT116 cells, some mTOR staining also overlaps with the *trans*-Golgi network (red; indicated by arrows in merged images). DAPI (4',6-diamidino-2-phenylindole) marks the nucleus in (**a**) (blue) and (**b**) (white). Scale bars are 5 μm. Confocal channels are indicated as follows in the merged images: green (G), blue (B) and red (R).

Immunofluorescence staining of HCT116 cells revealed that PAT4 is concentrated on and adjacent to the *trans*-Golgi network (Figure 7a). A similar localisation was observed in 786-O cells (Supplementary Figure S5A). A previous report also suggested that overexpressed PAT4 in HEK-293T cells is not on LELs.^32^ Interestingly, although under normal culture conditions, most mTOR is localised around the LELs, some colocalisation with the trans-Golgi network was also observed (Figure 7b), raising the possibility that it might be associated with PAT4 in this compartment.

**Figure 8:**
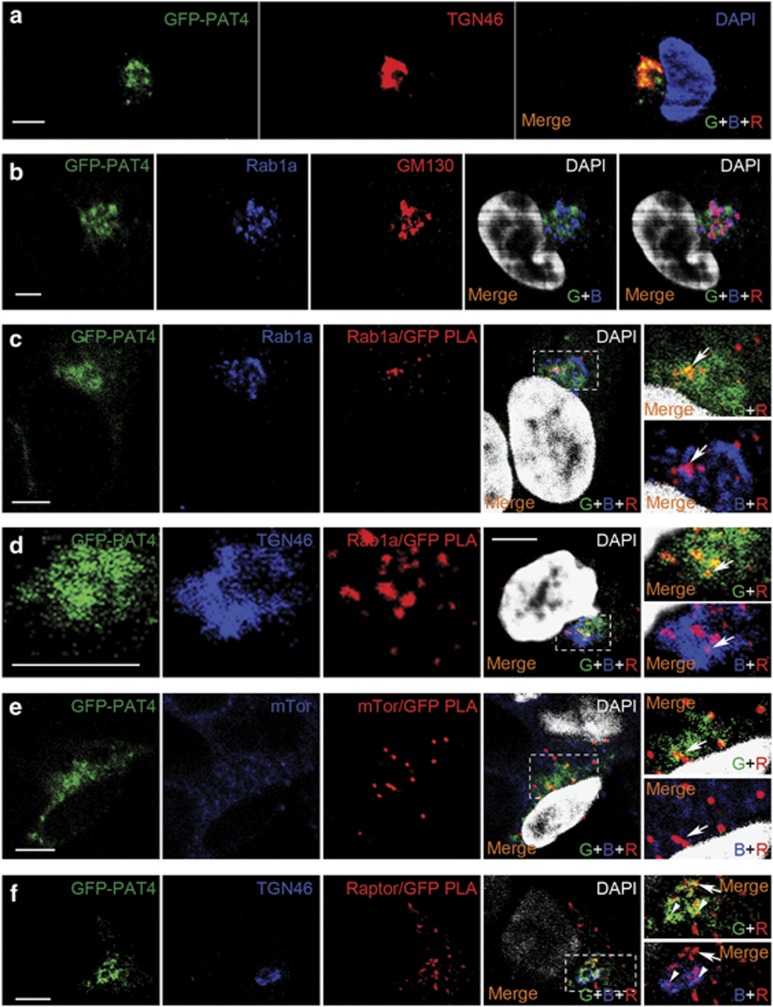
PAT4 interacts with Rab1A and mTORC1 on the Golgi. (**a**) GFP-PAT4 fusion protein (green) in HEK-293 cells shows a similar subcellular localisation to HCT116 cells on the *trans*-Golgi (TGN46; red). An alternative cell at higher resolution is stained with the same markers in (**d**). (**b**) Rab1A (blue) is localised primarily on the *cis*-Golgi, labelled by the GM130 marker (red). (**c**) *In situ* PLA (red) reveals interaction between Rab1A (blue) and GFP-PAT4 (green) primarily associated with GFP-PAT4-containing compartments (overlap between GFP-PAT4 and PLA signal is yellow in merge, top right panel; arrow). The PLA signal is also adjacent and partially overlapping with compartments on which Rab1A is concentrated in merge of Rab1A and PLA, bottom right panel. Rab1A/PAT4 PLA signals are only observed in GFP-PAT4-expressing cells (see Supplementary Figure S5B). (**d**) Some of the GFP-PAT4/Rab1a PLA-positive interacting compartments (red) also appear to be partly or entirely labelled by *trans*-Golgi network marker TGN46 (blue; arrow), but not *trans*-Golgi regions lacking GFP-PAT4. (**e**) PLA (red) reveals an interaction between mTOR (blue) and GFP-PAT4 (green) partially overlapping with GFP-PAT4-containing compartments (yellow in merges containing green and red channels, including magnified image in top right panel; arrow). In the low magnification merge image, the two upper cells not expressing GFP-PAT4 do not give a PLA signal, indicating that this assay specifically detects the GFP-PAT4/mTOR interaction. Blue and red channel merge (lower right-hand panel) reveals that mTOR staining is often in close proximity to PLA signal, but is also found in many other locations within the cell. (**f**) PLA (red) reveals an interaction (red) between Raptor and GFP-PAT4 (green), in and adjacent to compartments containing GFP-PAT4 (yellow overlap in merges containing green and red channels, including magnified image in top right panel; arrow and arrowheads). Blue and red channel merge (lower right-hand panel) reveals that some PLA signals are adjacent or partially colocalise with the *trans*-Golgi (TGN, blue; arrowheads), whereas others do not (arrow). Raptor/GFP-PAT4 PLA signals are only observed in GFP-PAT4-expressing cells (see Supplementary Figure S5C). DAPI marks the nucleus in (**a**) (blue) and (**b**–**f**) (white) in the merge. Confocal channels are indicated as follows in the merged images: green (G), blue (B) and red (R). Scale bars are 5 μm.

To investigate this further, we used rapamycin-resistant HEK-293 cells expressing GFP-tagged PAT4 (Figure 5d). GFP-PAT4 also localised on and around the *trans*-Golgi network (Figure 8a). Golgi-localised Rab1A, a monomeric GTPase involved in membrane trafficking events, has recently been implicated in amino-acid-dependent activation of mTORC1 from the Golgi.^20^ We tested whether Rab1A might interact with PAT4. We used the proximity ligation assay (PLA), which detects specific protein–protein interactions *in situ*, when antibodies recognising these molecules are in close proximity.^33^ Although anti-Rab1A staining primarily localised to the *cis*-Golgi (Figure 8b), Rab1A and GFP antibodies produced a PLA signal on an adjacent compartment (Figure 8c), which was not present in cells that did not express GFP-PAT4 (Supplementary Figure S5B). This signal frequently overlapped with GFP-PAT4 and was partly within the *trans*-Golgi network (Figure 8d), suggesting that Rab1A and PAT4 can interact on the Golgi. In addition, PLA using either anti-mTOR (Figure 8e) or anti-Raptor (Figure 8f and Supplementary Figure S5C) antibodies with anti-GFP also produced a specific signal primarily in GFP-PAT4-containing compartments that included the *trans*-Golgi network. This indicates that mTORC1 interacts with PAT4 on the Golgi, consistent with the idea that PAT4 can regulate mTORC1 activity from this compartment.

## Discussion

Although resistance of mTORC1 to inhibitors can be partly explained by differential *in vitro* sensitivity of substrate target sites,^34^ increasing evidence indicates there are also different mTORC1 complexes in cancer cells,^20, 21, 22, 23, 35^ which may make them differentially sensitive to drugs such as rapamycin. In this study, we demonstrate that PAT4 regulates rapamycin-resistant mTORC1 in HCT116 cells and can induce increased rapamycin resistance when overexpressed in HEK-293 cells. PAT4 and rapamycin-resistant mTORC1 are essential for normal cell proliferation *in vitro*. Furthermore, PAT4 expression levels are predictive of early relapse in colorectal cancer, suggesting a pathophysiological role in the acquisition of more aggressive tumour phenotypes.

**Figure 9:**
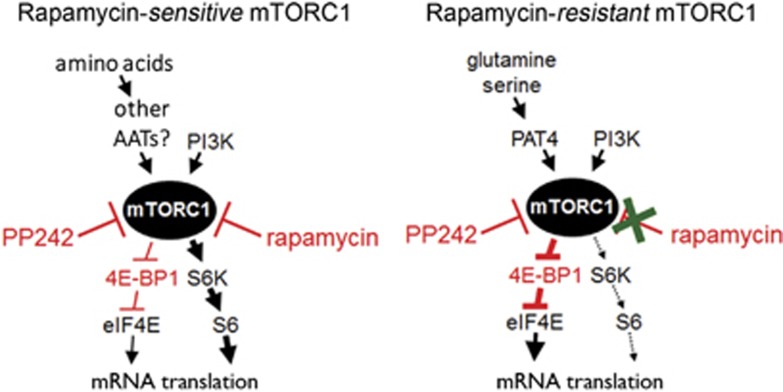
Schematic diagrams of rapamycin-sensitive and -resistant mTORC1 complexes in HCT116 cells. S6K and 4E-BP1, a negative regulator of eukaryotic initiation factor 4E(eIF4E), are the best characterised downstream targets of mTORC1. Although rapamycin treatment strongly inhibits S6K phosphorylation, it has a weaker effect on 4E-BP1 γ-band phosphorylation at Ser65. Less phosphorylated forms of 4E-BP1 bind to eIF4E leading to translational repression.^8^ Reducing PAT4 activity primarily affects the rapamycin-resistant form of mTORC1. This leads to a reduction in a Ser65-phosphorylated form of 4E-BP1, but has less effect on S6K phosphorylation. Other AATs in the SLC36 (PAT) and/or SLC38 family are likely to be involved in rapamycin-sensitive mTORC1 regulation, for example, PAT1 and SLC38A9. PP242 is an mTORC1 ATP-kinase inhibitor that acts on both the rapamycin-sensitive and -resistant forms of mTORC1. Arrows signify positive signals and cross-bars mark inhibitory signalling events.

Our findings support a model in which rapamycin-resistant and -sensitive forms of mTORC1 can be independently controlled, and provide a new genetic tool to separate these two signalling functions (Figure 9). Rapamycin-resistant mTORC1 selectively, but not exclusively, regulates 4E-BP1 hyperphosphorylation, which is also specifically targeted by P53 activation in murine erythroleukemia cells,^27^ further supporting the idea that it is a distinct complex. However, this selective effect (Figure 4 and Supplementary Figure S2C) is not universal. In other cell types, PAT4 primarily appears to regulate an mTORC1 complex controlling 4E-BP1 and S6K (Supplementary Figure S3; Heublein *et al.*^14^), suggesting that cell-type-specific regulatory factors or PAT4 expression levels modulate this transporter's specificity in controlling mTORC1. Indeed, it is only upon PAT4 overexpression that HEK-293 cells exhibit detectable PAT4-dependent rapamycin resistance (Figure 5d).

It has been suggested that PAT4 regulates mTORC1 signalling by transporting amino acids across the plasma membrane.^36^ However, this seems unlikely in our cell models for three reasons: first, cell surface PAT4 protein levels are very low (Figures 1a, 7 and 8);^32, 36^ second, PAT4 appears to be a low-capacity transporter;^37^ third, it is difficult to explain how a cell surface transporter might affect only rapamycin-resistant mTORC1. We favour a model where PAT4 interacts with mTOR on the Golgi, although it may also regulate mTORC1 from other subcellular compartments.

Our study also revealed selective sensitivity of the mTORC1 target 4E-BP1 in HCT116 cells to reduction of two non-essential amino acids, glutamine and serine, but not to the mTORC1 regulator leucine, whose intracellular levels can be modulated by glutamine.^38^ The Arf1 GTPase, which regulates trafficking in multiple compartments, including the Golgi, has also recently been implicated in glutamine sensing by mTORC1.^22^ A key question is whether PAT4 and Arf1 are involved in a common mTORC1-regulatory mechanism. As PAT4's amino-acid specificity is different to arginine-sensitive, mTORC1-regulatory SLC38A9^18, 19^ and PAT1,^15^ it seems likely that a range of AATs may determine the sensitivity of mTORC1 to different amino acids. The unique N-terminal domain of SLC38A9 appears to bind the Ragulator complex with higher affinity compared with other transporters.^18^ SLC38A9 also interacts at lower affinity with the vacuolar-H^+^-ATPase through sequences including its transmembrane domains, which share similarity with other SLC38 and SLC36 (PAT) family members. This may explain why several of these transporters can regulate mTORC1, but some, such as PAT4, cannot pull down other mTORC1 supercomplex components, even though PLA indicates they interact *in situ*.

The PAT4-dependent sensitivity of 4E-BP1 hyperphosphorylation to 4 h of glutamine or serine starvation probably reflects the rapid metabolism of these two non-essential amino acids in HCT116 cells.^23, 27^ Despite its name, when heterologously expressed in *Xenopus* oocytes, PAT4 can transport amino acids via a non-proton-coupled mechanism. It appears to have a very high substrate affinity, but low capacity, for proline and tryptophan.^37^ Several other amino acids, including glutamine and serine, bind with lower affinity, and can compete with high-affinity PAT4 substrates, although they may not be transported. This could provide a transport-independent, amino-acid-sensing ‘transceptor' mechanism, in keeping with previous suggestions for the PATs.^17^ Cells would become more resistant to depletion of highly metabolised amino acids by expressing more PAT4, explaining the clinical data from colorectal cancer patients.

In conclusion, our study suggests that pharmacological inhibition of an upstream mTORC1 activator such as PAT4 in colorectal cancer could complement the actions of rapamycin, by blocking a rapamycin-resistant, 4E-BP1-selective pathway. PAT4 may also provide a new biomarker for more aggressive colorectal tumours that are rapamycin-resistant.

## Materials and methods

### Cell culture

HCT116 cells were cultured in McCoy's 5A modified medium (Gibco, Invitrogen, Paisley, UK) containing 10% foetal calf serum (Gibco, Invitrogen), unless otherwise specified. The 786-O and HEK-293 cells were cultured in 10% foetal calf serum-containing Dulbecco's modified Eagle's medium. Cells were incubated at 37°C in 5% CO_2_. Rapamycin treatment was carried out 24 h after seeding. Cells were treated for 24 h typically, or 72 h for cell proliferation analysis, with 3 nM (HCT116) or 100 nM (HEK-293) rapamycin, diluted from a stock solution in dimethyl sulfoxide (R8781; Sigma, Gillingham, UK), or as specified for dose–response curves. Founder cell lines were obtained from ATCC (LGC Standards, Teddington, UK) and used within 6 months of resuscitation (<25 passages). si435 used to knockdown *PAT4* expression and the scrambled control siRNA (Figure 1) were previously described in Heublein *et al.*^14^

### Inducible shRNA-expressing lentiviruses

Sigma Mission lentiviral particles carrying the following clones: TRCN0000043984, 5′-CCGGCCTTGATAAATGAGCAGAATTCTCGAGAATTCTGCTCATTTATCAAGGTTTTTG-3′ (referred to as shPAT4(43 984)); TRCN0000043985, 5′-CCGGCCTGGAGAGTAAAGTGTTTATCTCGAGATAAACACTTTACTCTCCAGGTTTTTG-3′ (shPAT4(43 985)) and TRCN0000043987, 5′-CCGGCCAGTATGTTGTCAGGAACATCTCGAGATGTTCCTGACAACATACTGGTTTTTG-3′ (shPAT4(43 987)) and a non-targeting control construct (SHC312V; shNT) in the IPTG-inducible lentiviral vector pLKO IPTG_1xLacO were transduced into HCT116 cells. Although shPAT4(43 984) partially overlaps with siRNA(435), shPAT4(43 985) and (43 987) have no overlap with previously used siRNAs.^14^ For most experiments, clone shPAT4(7.1) produced a stronger knockdown compared with clone shPAT4(4.8), with greater effects on proliferation and mTORC1 signalling.

### Generation and induction of shRNA lines

Cells were transduced with lentiviral vectors at a multiplicity of infection of three (viral particles to cells) in the presence of polybrene. Puromycin selection began 2 days after transduction to generate pools of cells derived from multiple transduction events. Single-cell clones were isolated soon after transduction using the limiting dilution method. IPTG (100 μm) was added for induction. IPTG-treated cells were preinduced for 5 days before plating.

### Amino-acid starvation media

Medium was based on Dulbecco's modified Eagle's medium (11995-065; Invitrogen), but made in-house, so that different amino acids could be omitted individually. For glutamine, a medium lacking this amino acid was already available (McCoy's 5A without glutamine (M8403; Sigma)). Cells were starved of amino acids for 4 h.

### Generation of GFP-PAT4 stable cell line

The PAT4 open reading frame from IMAGE Clone ID:531323 was recombined into a pOPINE vector containing an in-frame C-terminal GFP sequence (pOPINE-GFPc; gift from J Beale and S Newstead). The following errors in the *PAT4* cDNA sequence (compared with the annotated transcript sequence) were corrected using the Quickchange Site-Directed Mutagenesis Kit (Invitrogen): I209L (ATA->CTA) H376P (CCT->CAT) I429L (ATA->CTA). The PAT4-GFP fusion was amplified by PCR and cloned into pcDNA3.1(+) at the *Kpn*I and *Xba*I sites. Amplified PAT4 and GFP open reading frames were recloned into pcDNA3.1(+) as a GFP-PAT4 fusion. HEK-293 cells were transfected with this construct or the empty pcDNA3.1(+) vector using Lipofectamine 2000 (Invitrogen). Stably transfected cells were selected 48 h later using 800 μg/ml Geneticin (Gibco, Invitrogen), as described in Heublein *et al.*^14^

### Xenograft studies

All protocols were carried out under Project Licence 30/2771 following Home Office regulations^39^ using 6- to 7-week-old female BALB/c SCID *nu*/*nu* mice (Harlan Sprague Dawley Inc., Bicester, UK). A total of 2.5 × 10^6^ HCT116 cells in 50 μl serum-free medium and 50 μl Matrigel (BD Bioscience, Oxford, UK) were subcutaneously injected into one flank (seven mice per group; animals not randomized and investigator not blinded; numbers were based on previous xenograft studies). In all, 10 mM IPTG (Carbosynth Inc., Compton, UK) was added to drinking water of treated mice. Tumour growth was measured three times a week using callipers and volume calculated from the formula 1/6π x length x width x height. The experiment was repeated and produced similar significant reductions in growth and effects on survival time.

### Quantitative real-time PCR

RNA extraction and quantitative real-time PCR were carried out as described previously,^14^ with Ct values for *PAT4* normalised against the *HPRT1* housekeeping gene.

### Cell proliferation analysis

Cell proliferation experiments, each using a minimum of three wells, were repeated three times. Where statistically significant results were found, these were observed on all occasions. Cells were counted according to the methodology described in Heublein *et al.*^14^

### Western blots

Protein lysates were routinely loaded on 10% polyacrylamide gels. For immunoblotting, the following primary antibodies were used at recommended dilutions: phospho-S6K1 Thr308 (Cell Signalling Technology, Hitchin, UK (CST; no.9205)), S6K1 (CST; no. 9202), phospho-Ser240/244-ribosomal S6 protein (CST; no. 2215)), S6 ribosomal protein (CST; no. 2217), phospho-Ser65-4E-BP1 rabbit polyclonal (CST; no. 9451), 4E-BP1 (CST; no. 9644), phospho-Ser473-Akt (CST; no. 4060), Akt (CST; no. 9272), phospho-Thr202/Tyr204-p44/42 mitogen-activated protein kinase (Erk1/2; CST; no. 4370), p44/42 mitogen-activated protein kinase (Erk1/2; CST; no. 4695) and α-tubulin (Sigma; no. T6199). They were detected with secondary antibodies (Promega, Southampton, UK; nos. W401B and W402B) and visualised using the SuperSignal West Pico Chemiluminescent Substrate Kit (Thermoscientific, Loughborough, UK). PAT4 was deglycosylated with PNGase F (New England Biolabs, Hitchin, UK; no. P0704) according to the manufacturer's instructions.

### Generation of PAT4 monoclonal antibody

A monoclonal antibody against PAT4 was created by immunising mice with a keyhole limpet haemocyanin-conjugated, cysteine-coupled peptide (Severn Biotech Ltd., Kidderminster, UK) based on an antigenic amino-acid sequence within the N terminus of PAT4 (REELDMDVMRPLINE-C).

### Immunohistochemistry

The following commercial primary antibodies were used: mTOR (CST; no. 2983; 1/100), LAMP2 mouse monoclonal (Abcam, Cambridge, UK; ab25631; 1/100), TGN46 sheep polyclonal (Novus Biologicals, Abingdon, UK; NB110-40767: 1/200), Rab1a rabbit monoclonal (CST; no. 13075; 1/100), GFP mouse monoclonal (Abcam; ab1218; 1/100), GM130 mouse polyclonal (Novus Biologicals; H00002801-B01P; 1/25), Raptor rabbit polyclonal (Merck Millipore, Watford, UK; 09-217; 1/100) and secondary antibodies raised in donkey (Jackson ImmunoResearch, West Grove, PA, USA; 1/500). Cells were processed and imaged as described in Ogmundsdottir *et al.*^13^ and Sancak *et al.*^40^ PLAs were performed using the Duolink *In Situ* Orange Starter Mouse/Rabbit Kit (DUO92102; Sigma) according to the manufacturer's instructions.

For patient samples, slides were dewaxed and rehydrated with antigen retrieval performed using citrate buffer pH 6.0 (Sigma-Aldrich, Gillingham, UK) and a pressurised decloaking chamber (Biocare Medical, Concord, CA, USA). The intensity of cytoplasmic staining was assessed by a pathologist (CS) on a semiquantitative scale from 0 to 3. High levels of expression in tumour sections were scored 3, and lower levels of expression scored 0, 1 or 2.

Immunohistochemistry was carried out as described previously.^41^ Paraffin-embedded tissue blocks from formalin-fixed tumour samples were sectioned, dewaxed and rehydrated using standard techniques and 4 μm sections.

### Patient material

Formalin-fixed and paraffin-embedded tissue was obtained with informed consent from 111 patients with colon cancer treated by surgical resection at the John Radcliffe Hospital (Oxford, UK) from 1997 to 2000. The sample size was limited to 107 by availability of tissue with full clinicopathological data, follow-up data and ethical approval. Use of tissue in this project was approved by the Oxford Centre for Histopathological Research Panel (Project number 12/A172) and the local Research Ethics Committee (C02.216). No patients received preoperative chemotherapy. The average age at the time of surgery was 71 years (range 37–96 years), 64 patients were male (58%) and the average follow-up time was 4.1 years (as of January 2009). Based on the tumour, nodal and distant metastases, cancer stage of patients at resection was as follows: 10 patients (9%) stage 1; 55 patients (49%) stage 2; 35 patients (32%) stage 3 and 11 patients (10%) stage 4. All resections resulted in clear margins. Relapse-free survival was defined as time between tumour resection and the first documented recurrence of tumour at any site. Patients who died from unrelated causes were excluded. Tissue microarrays were assembled as described previously^42^ using two representative cores of tumour and two representative cores of adjacent normal colonic mucosal epithelium for each patient.

### Statistics

All data (means±s.e.m. (xenografts) or s.d (*in vitro*)) were analysed using Excel or GraphPad Prism 5 (GraphPad Software, La Jolla, CA, USA). For tumour growth analysis, a parametric generalised linear model was performed using GraphPad Prism 4.0b software. For western blot analysis, cell proliferation and quantitative real-time PCR, statistical significance was determined using the Kruskal–Wallis one-way analysis of variance. All *in vitro* experiments were replicated on at least three occasions. For patient data, the *χ*^2^ test was used to determine association between PAT4 expression and categorical clinical variables (Supplementary Table S1). Cox regression analysis was used to determine prognostic factors in univariate (Supplementary Table S2) and multivariate survival models (Supplementary Table S3). The log-rank (Mantel–Cox) test was used to assess the significance of differences in relapse-free survival between Kaplan–Meier curves. Statistical analyses were performed using SPSS Statistics (Version 21.0, IBM, New York, NY, USA).
