# Supplementary figures and images for: PAT4 levels control amino-acid sensitivity of rapamycin-resistant mTORC1 from the Golgi and affect clinical outcome in colorectal cancer
Source: Oncogene. 2015 Oct 5;35(23):3004–15. doi: 10.1038/onc.2015.363 (PMC4705441; doi:10.1038/onc.2015.363)

Figure S1

A

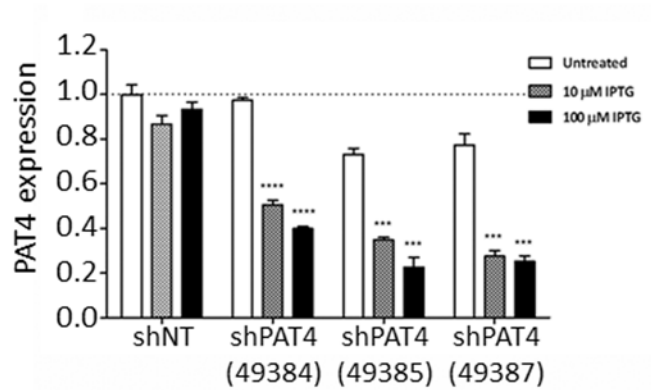

B

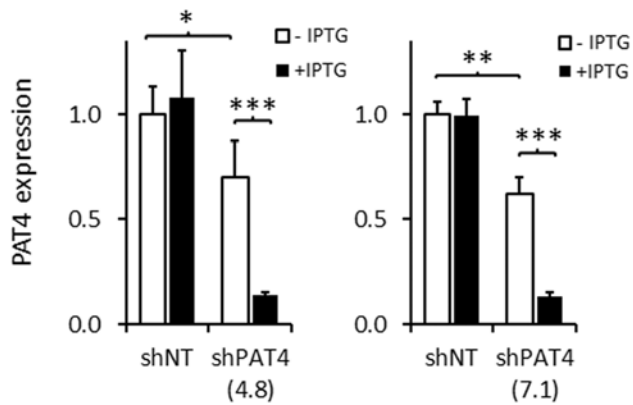

Supplement: Supplementary Figure S1 [file onc2015363x1.pdf]

Figure S2

A

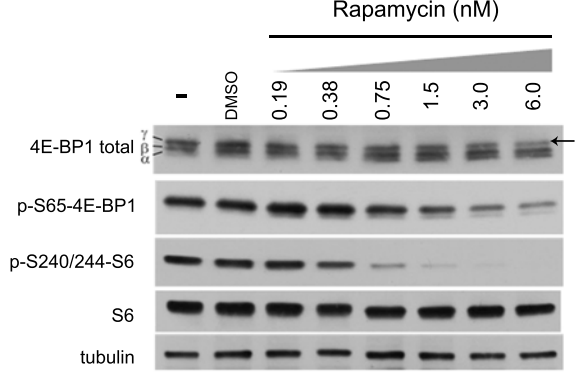

B

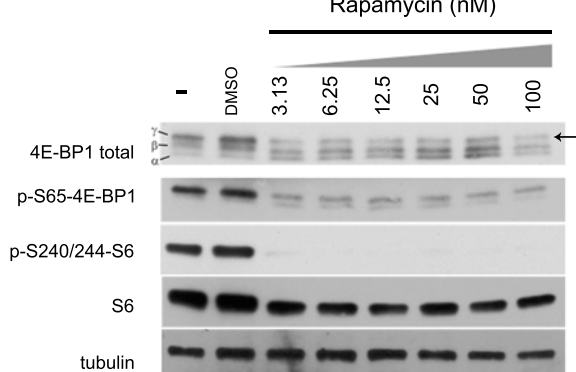

E

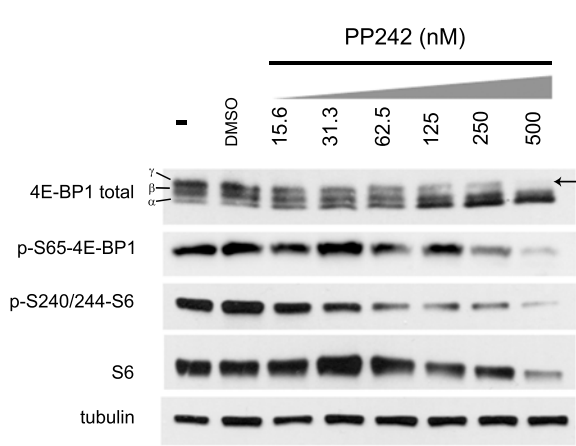

C

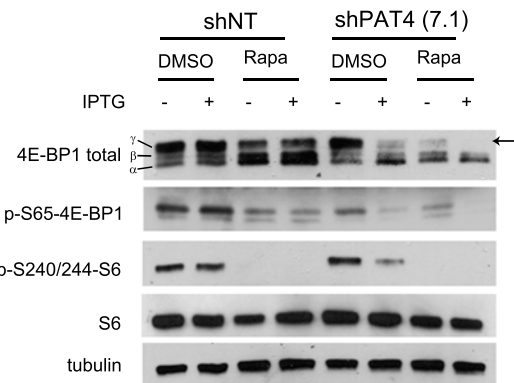

D

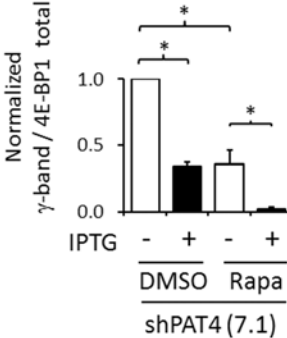

F

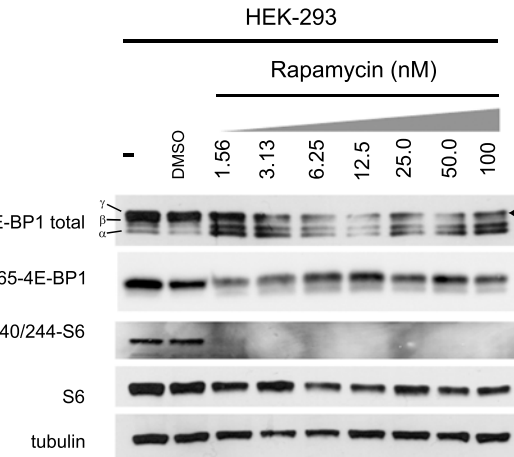

Supplement: Supplementary Figure S2 [file onc2015363x2.pdf]

Figure S3

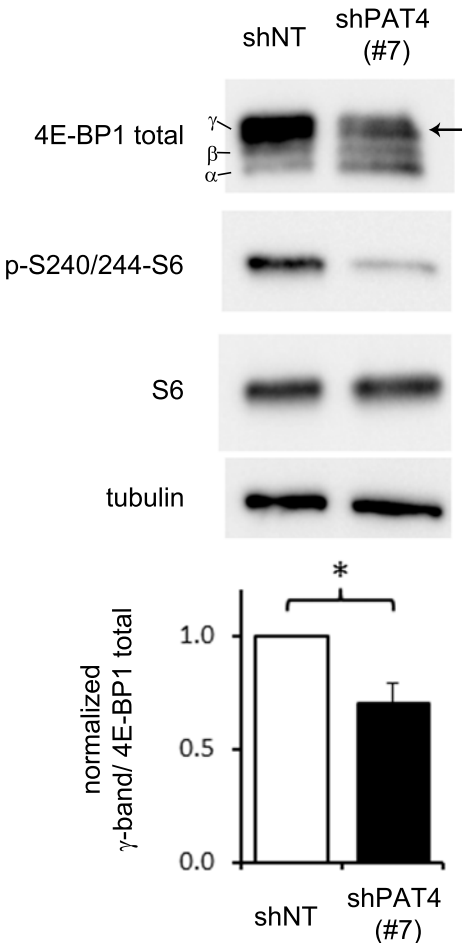

Supplement: Supplementary Figure S3 [file onc2015363x3.pdf]

Figure S4

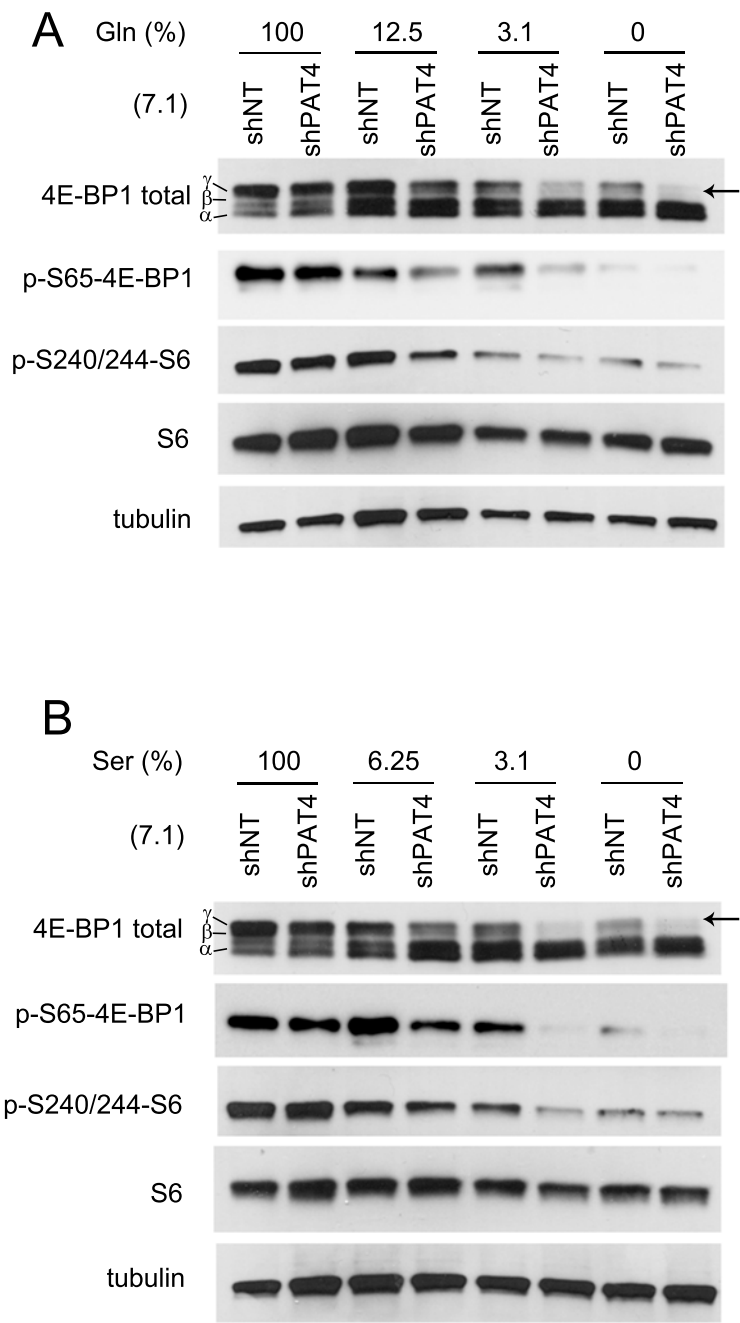

Supplement: Supplementary Figure S4 [file onc2015363x4.pdf]

Figure S5

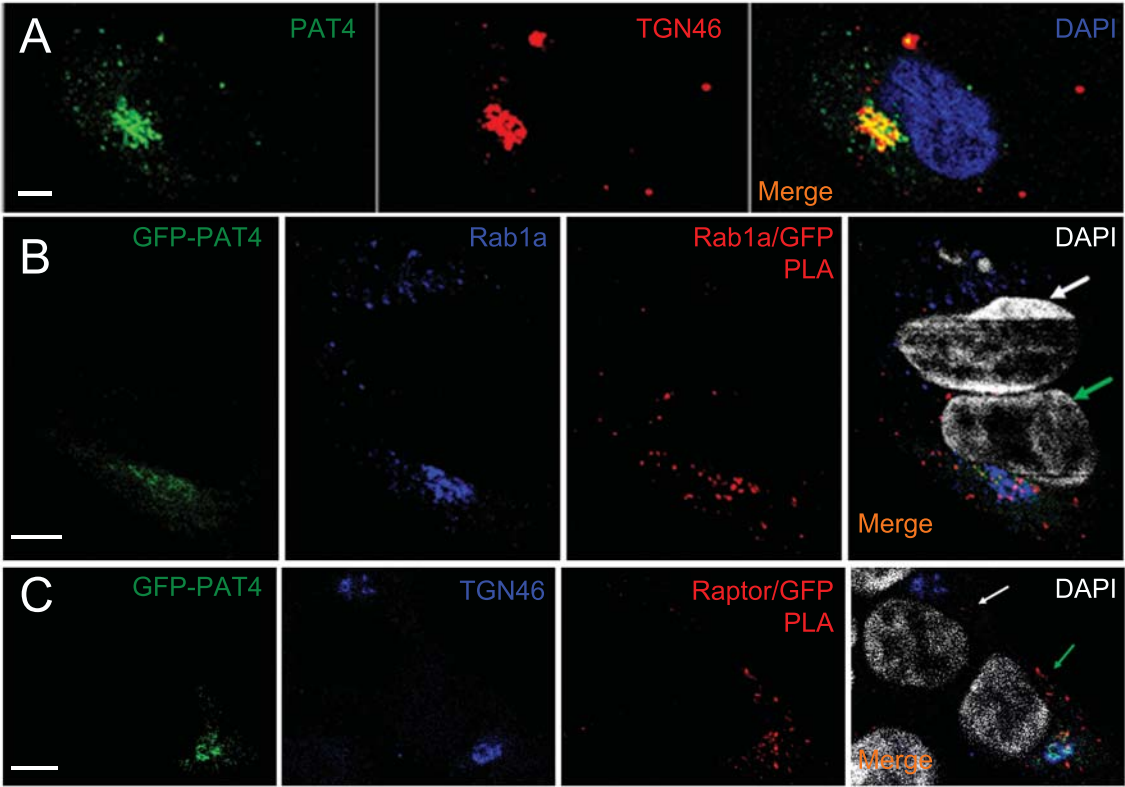

Supplement: Supplementary Figure S5 [file onc2015363x5.pdf]
