# Supplementary material for: PAT4 levels control amino-acid sensitivity of rapamycin-resistant mTORC1 from the Golgi and affect clinical outcome in colorectal cancer
Source: Oncogene. 2015 Oct 5;35(23):3004–15. doi: 10.1038/onc.2015.363 (PMC4705441; doi:10.1038/onc.2015.363)
Supplement: Supplementary Figure Legends [file onc2015363x6.docx]

**Supplementary Data**

**Supplementary Figure Legends**

**Supplementary Figure S1.** *PAT4* expression is strongly reduced following IPTG-induced *PAT4* shRNA knockdown in HCT116 cells. **A**, *PAT4* expression was measured using quantitative real-time PCR of RNA preparations from HCT116 cells carrying three different pools of IPTG-inducible *PAT4* shRNAs, shPAT4(43984), shPAT4(43985) and shPAT4(43987), as well as a control non-targeting shRNA construct (shNT). The effects of 10 μM and 100 μM IPTG treatment compared to untreated cells after five days of culture were measured. **B**, *PAT4* expression was measured from shPAT4(4.8) or shPAT4(7.1), two independent HCT116 cell clones, each carrying an IPTG-inducible PAT4 shRNA. The effect of IPTG induction with 100 μM IPTG was compared to non-induced cells and also HCT116 cells carrying shNT. Leaky PAT4 shRNA expression was observed in non-induced HCT116 cells carrying the IPTG-inducible *PAT4*-shRNA constructs; *P<0.05 (n=3).

**Supplementary Figure S2.** Rapamycin-resistant mTORC1 in HCT116 and HEK-293 cells and the role of PAT4. **A** and **B***,* dose-response analysis of HCT116 cells using a low (A) and high (B) range of rapamycin concentrations. 4E-BP1 has eight or more phosphorylation sites (1), which produce modified proteins that migrate differently on polyacrylamide gels. These can be resolved into three bands (α, β and γ), with the most phosphorylated form being the γ-band. 4E-BP1 protein in the γ-band is typically phosphorylated at Ser65 (Fig. 4C and D). Prolonged rapamycin treatment has a pronounced dose-dependent effect on phospho-S6 (p-S240/244-S6), but only partially reduces the levels of the 4E-BP1 γ-band (arrow in 4E-BP1 total), even at the highest rapamycin concentrations (B). Some phospho-Ser65-containing 4E-BP1 (p-S65-4E-BP1) runs at a lower molecular weight, suggesting that rapamycin in part reduces the γ-band by decreasing modification at one or more other undefined phosphorylation sites on 4E-BP1. **C** and **D**, stably transfected HCT116 cells carrying the IPTG-inducible *PAT4* knockdown construct, shPAT4(7.1), or the IPTG-inducible non-targeting control, shNT, were cultured for five days in the absence or presence of IPTG and if required, treated with 3 nM rapamycin for the last 24 h. Rapamycin strongly reduces phospho-S6 levels (p-S240/244-S6), and partially affects the hyperphosphorylated 4E-BP1 γ-band. The rapamycin-resistant γ-band is lost after *PAT4* knockdown (D; *P<0.05). The blot was also probed with pan-S6 and anti-tubulin antibodies as a loading control. **E**, treatment of HCT116 cells with the mTOR ATP kinase inhibitor, PP242, for 24 h strongly inhibits the formation of both phospho-S6 (p-S240/244-S6), and the γ-phosphorylated form of 4E-BP1, consistent with an effect on both rapamycin-sensitive and -resistant forms of mTORC1. **F**, dose-response analysis of HEK-293 cells reveals they are also show resistant to rapamycin treatment. There is only a partial reduction in the 4E-BP1 γ-band (arrow in 4E-BP1 total), even at the highest rapamycin concentrations tested.

**Supplementary Figure S3**. PAT4 regulates 4E-BP1 and S6 phosphorylation in HEK-293 cells. Western blots of protein extracts from HEK-293 cells transfected with constructs carrying either a constitutively expressed, non-targeting shNT control or *PAT4* knockdown gene, shPAT4(43987; #7). *PAT4* knockdown significantly reduced the level of the most phosphorylated γ-form of 4E-BP1 and phospho-S6 (p-S240/244-S6), consistent with previous siRNA knockdown data (2). Blots were probed with an anti-tubulin antibody as a loading control. *P<0.05, n=3.

**Supplementary Figure S4**. Reduced levels of PAT4 make rapamycin-resistant mTORC1 signalling more sensitive to glutamine and serine depletion in HCT116 cells with a second independent shPAT4 clone. **A** and **B**, stably transfected HCT116 cells carrying the IPTG-inducible, *PAT4* knockdown construct, shPAT4(7.1), or the IPTG-inducible non-targeting control, shNT, were exposed to culture medium containing different concentrations of glutamine (A) and serine (B) for four hours in the absence of IPTG. Under these conditions, due to leaky expression of the construct, shPAT4(7.1) cells express about 50% of normal *PAT4* mRNA levels (Supplementary Fig. S1B). γ-phosphorylation of 4E-BP1 (arrow) is reduced in shPAT4(7.1) cells at lower concentrations of glutamine and serine. As is the case for shPAT4(4.8) (Fig. 6B and C), a minor effect on p-S240/244-S6 levels is also typically observed in *PAT4* knockdown cells at low amino acid concentrations, perhaps because PAT4-dependent mTORC1 also has a modest effect on S6K activation.

**Supplementary Figure S5.** PAT4 is localised on the Golgi and specifically interacts with Rab1A and Raptor. **A**, the subcellular localisation of PAT4 in 786-O cells was analysed by confocal microscopy using the PAT4 monoclonal antibody. PAT4 (green) predominantly co-localises with the trans-Golgi marker TGN46 (red). **B**, specific interaction between GFP-PAT4 (green) and Rab1A (blue) was detected by *in situ* proximity ligation assay (PLA; red) in HEK-293 cells expressing GFP-PAT4 (green arrow), but not in cells lacking this fusion protein (white arrow). **C**, specific interaction between GFP-PAT4 (green) and Raptor was detected by *in situ* proximity ligation assay (PLA; red) in the trans-Golgi (blue) and other compartments in HEK-293 cells expressing GFP-PAT4 (green arrow), but not in cells lacking this fusion protein (white arrow). In the merged images (Merge), DAPI marks the nucleus, blue (A) or white (B, C). Scale bars are 5 µm.

**SUPPLEMENTARY REFERENCES**

[1] Constantinou C, Clemens MJ. (2005) Regulation of the phosphorylation and integrity of protein synthesis initiation factor eIF4GI and the translational repressor 4E-BP1 by p53. Oncogene 24, 4839-50.

[2] Heublein S, Kazi S, Ogmundsdottir MH, Attwood EV, Kala S, Boyd CA, Wilson C, Goberdhan DC (2010) Proton-assisted amino-acid transporters are conserved regulators of proliferation and amino-acid-dependent mTORC1 activation. Oncogene 29, 4068-79.
