# Supplementary material for: PAT4 levels control amino-acid sensitivity of rapamycin-resistant mTORC1 from the Golgi and affect clinical outcome in colorectal cancer
Source: Oncogene. 2015 Oct 5;35(23):3004–15. doi: 10.1038/onc.2015.363 (PMC4705441; doi:10.1038/onc.2015.363)
Supplement: Supplementary Tables [file onc2015363x7.docx]

| **Variable** | **χ^2^** | **P-value** |
| --- | --- | --- |
| **Age** | 98.90 | 0.40 |
| **Gender** | 1.44 | 0.23 |
| **Histologic differentiation** | 6.62 | 0.09 |
| **Overall stage** | 1.09 | 0.78 |
| **Bowel perforation** | 1.12 | 0.29 |
| **Obstruction** | 0.72 | 0.40 |
| **Lymphatic invasion** | 0.65 | 0.42 |
| **Vascular invasion** | 0.03 | 0.87 |
| **Neural invasion** | 0.07 | 0.79 |
| **Tumour stage (T)** | 2.99 | 0.39 |
| **Nodal metastases (N)** | 2.55 | 0.28 |
| **Metastases (distant; M)** | 0.001 | 0.98 |

**Table S1.**  Statistical significance of associations between high level PAT4 expression and categorical clinical variables. High level PAT4 expression is independent of all the other factors tested.

| **Variable** | **Category** | **n** | **Events** | **Hazard ratio** | **95% confidence interval** | **P-value** |
| --- | --- | --- | --- | --- | --- | --- |
| **PAT4** | Low (1, 2)  High (3) | 58  49 | 4  13 | 4.39 | 1.43 - 13.40 | **0.01** |
| **Age** | <60  >60 | 21  90 | 5  14 | 0.64 | 0.23 - 1.76 | 0.44 |
| **Gender** | Female  Male | 48  63 | 10  9 | 0.68 | 0.28 - 1.68 | 0.41 |
| **Differentiation** | Well Moderate Poor | 6  86  16 | 0  15  4 | 8,830  12,800 | 0.00 - 8.40 x 10^131^  0.00 - 1.18 x 10^110^ | 0.94  0.94 |
| **Stage**  **(1/2 or 3/4)** | 1,2  3,4 | 65  46 | 5  14 | 4.40 | 1.58 - 12.20 | **<0.01** |
| **Bowel perforation** | No Yes | 105  6 | 16  3 | 4.22 | 1.23 - 14.50 | **0.02** |
| **Obstruction** | No  Yes | 106  5 | 18  1 | 1.10 | 0.15 - 8.21 | 0.93 |
| **Lymphatic invasion** | No Yes | 81  30 | 12  7 | 1.65 | 0.65 - 4.18 | 0.29 |
| **Vascular invasion** | No  Yes | 72  39 | 11  8 | 1.40 | 0.56 - 3.48 | 0.47 |
| **Neural invasion** | No  Yes | 106  5 | 16  3 | 5.41 | 1.57 - 18.60 | **<0.01** |
| **Tumour stage (T; 1/2/3 or 4)** | 1, 2, 3  4 | 76  35 | 7  12 | 4.34 | 1.71 - 11.00 | **<0.01** |
| **Nodal metastases (N)** | 0  1, 2 | 66  45 | 6  13 | 3.49 | 1.33 - 9.18 | **<0.01** |
| **Metastases** **(distant; M)** | No  Yes | 96  15 | 13  6 | 3.61 | 1.37 - 9.50 | **<0.01** |

**Table S2.** Univariate analysis of relapse-free survival (Cox regression analysis). High level PAT4 expression is associated with shorter relapse-free survival.

| **Variable** | **Hazard ratio** | **95% confidence interval** | **Association with shorter relapse-free survival** | **P-value** |
| --- | --- | --- | --- | --- |
| **PAT4** | 5.69 | 1.70 - 18.80 | Higher expression | **<0.01** |
| **Bowel perforation** | 1.39 | 0.35 - 5.57 |  | 0.64 |
| **Neural invasion** | 4.61 | 1.14 - 20.16 | Neural invasion | **0.03** |
| **Tumour stage (T)** | 2.46 | 0.80 - 7.60 |  | 0.11 |
| **Nodal metastases (N)** | 2.32 | 0.73 - 7.43 |  | 0.16 |
| **Metastases (distant; M)** | 1.79 | 0.57 - 5.70 |  | 0.32 |

**Table S3**. Multivariate analysis of relapse-free survival (Cox regression analysis). High level PAT4 expression is associated with shorter relapse-free survival.

|  | **Percentage of dead or damaged cells** | | |
| --- | --- | --- | --- |
| **Sample** | **Day 1** | **Day 2** | **Day 3** |
| **shNT - IPTG** | 0.87% ± 0.90% | 0.81% ± 0.45% | 0.92% ± 0.37% |
| **shNT + IPTG** | 0.63% ± 0.24% | 1.30% ± 0.53% | 0.83% ± 0.48% |
| **shPAT4(4.8) - IPTG** | 0.67% ± 0.32% | 1.56% ± 0.81% | 1.82% ± 1.54% |
| **shPAT4(4.8) + IPTG** | 1.96% ± 1.30% | 1.95% ± 0.92% | 1.42% ± 0.56% |
| **shPAT4(7.1) - IPTG** | 1.33% ± 1.24% | 1.28% ± 0.66% | 1.24% ± 0.35% |
| **shPAT4(7.1) + IPTG** | 2.67% ± 0.82% | 1.59% ± 0.93% | 3.32% ± 1.36% |

**Table S4**. Analysis of HCT116 cell survival in *PAT4* knockdown experiments. Knockdown of *PAT4* in two different inducible knockdown clones [shPAT4(4.8) + IPTG and shPAT4(7.1) + IPTG] does not significantly alter cell survival, as measured by trypan blue staining of HCT116 cells under normal (10%) serum conditions. Statistical significance was determined using the Kruskal-Wallis one-way analysis of variance (P>0.1 for all conditions).
